# Acute sensitivity of the oral mucosa to oncogenic *K-ras*

**DOI:** 10.1002/path.2853

**Published:** 2011-03-07

**Authors:** Louise van der Weyden, Maria P Alcolea, Philip H Jones, Alistair G Rust, Mark J Arends, David J Adams

**Affiliations:** 1 Experimental Cancer Genetics, Wellcome Trust Sanger Institute, Wellcome Trust Genome CampusHinxton, CB10 1HH, UK; 2 MRC Cancer Cell Unit, Hutchison-MRC Research CentreCambridge, CB2 0XZ, UK; 3 Department of Pathology, Addenbrooke's Hospital, University of CambridgeCambridge, CB2 2QQ, UK

**Keywords:** oral mucosa, hyperplasia, carcinoma, *K*-*ras*, pAKT, pERK, cyclin D1

## Abstract

Mouse models of cancer represent powerful tools for analysing the role of genetic alterations in carcinogenesis. Using a mouse model that allows tamoxifen-inducible somatic activation (by Cre-mediated recombination) of oncogenic *K*-*ras*^*G*12*D*^ in a wide range of tissues, we observed hyperplasia of squamous epithelium located in moist or frequently abraded mucosa, with the most dramatic effects in the oral mucosa. This epithelium showed a sequence of squamous hyperplasia followed by squamous papilloma with dysplasia, in which some areas progressed to early invasive squamous cell carcinoma, within 14 days of widespread oncogenic *K*-*ras* activation. The marked proliferative response of the oral mucosa to *K*-*ras*^*G*12*D*^ was most evident in the basal layers of the squamous epithelium of the outer lip with hair follicles and wet mucosal surface, with these cells staining positively for pAKT and cyclin D1, showing Ras/AKT pathway activation and increased proliferation with Ki-67 and EdU positivity. The stromal cells also showed gene activation by recombination and immunopositivity for pERK indicating *K*-Ras/ERK pathway activation, but without Ki-67 positivity or increase in stromal proliferation. The oral neoplasms showed changes in the expression pattern of cytokeratins (CK6 and CK13), similar to those observed in human oral tumours. Sporadic activation of the *K*-*ras*^*G*12*D*^ allele (due to background spontaneous recombination in occasional cells) resulted in the development of benign oral squamous papillomas only showing a mild degree of dysplasia with no invasion. In summary, we show that oral mucosa is acutely sensitive to oncogenic *K*-*ras*, as widespread expression of activated *K*-*ras* in the murine oral mucosal squamous epithelium and underlying stroma can drive the oral squamous papilloma–carcinoma sequence. Copyright © 2011 Pathological Society of Great Britain and Ireland. Published by John Wiley & Sons, Ltd.

## Introduction

*Ras* genes are some of the most widely studied cancer-related genes, due to their frequent activation in human tumours 1. These small GTPases act as molecular switches that transfer information from the membrane to the nucleus. Ras proteins interact with multiple downstream effector pathways, the best studied of which are those involved in mitogenic signalling, such as the RAF/MEK/ERK pathway, and survival, such as the phosphoinositol-3 kinase (PI3K)/PDK/AKT pathway 2. Among *ras* oncogenes, *K*-*ras* is the most frequently activated in human tumours, with missense mutations at codons 12, 13, and 61 resulting in decreased GTPase activity and constitutive signalling 3. Although in some countries, such as India, the prevalence of *K*-RAS mutations in oral squamous cell carcinomas is as high as 50%, due to environmental factors, in Western countries such as the UK the prevalence is much lower, ∼5% 4–6. Following analysis of micro-RNA expression patterns in human oral squamous cell carcinomas (OSCC), Shin *et al* 6 concluded that miR-181a functions as an OSCC suppressor by targeting the *K*-RAS oncogene, suppressing expression of mutant *K*-RAS in OSCC cell lines and inhibiting their growth in culture, indicating the importance of mutated *K*-RAS as a driver mutation for OSCC growth. However, it remains to be established how mutated *K*-*ras* contributes to oral carcinogenesis in this small proportion of cases.

Unveiling the role of oncogenic *K*-*ras* in the development of human cancer has been greatly aided by the ability to manipulate the mouse genome in order to develop tumour models of sporadic *K*-*ras* activation, which closely recapitulate those events responsible for human disease 7–14. Indeed, the Jackson *et al* allele (*LSL-K-ras*^*G*12*D*^) 8 has been widely used to identify the effects of oncogenic *K*-*ras* in specific tissues, including the oral cavity 15, lung and GI tract 16, the haematopoietic system, 17, 18 and pancreas 16, 19, 20. This has been achieved by breeding *LSL-K-ras*^*G*12*D*^ mice with mouse lines that express Cre from tissue-specific inducible promoters.

In the present study, we bred *LSL-K-ras*^*G*12*D*^ mice with a mouse line carrying an inducible Cre recombinase (*Rosa*^*CreERT*2^). Administration of tamoxifen to these mice results in somatic expression of oncogenic *K*-*ras* in a wide variety of tissues. We observed hyperplasia of moist or frequently abraded squamous mucosa, with the most dramatic effects seen in the oral cavity: squamous papillomas showing dysplasia with areas of focal invasion (squamous cell carcinoma). Alternatively, sporadic expression of the *K*-*ras*^*G*12*D*^ allele (due to spontaneous recombination) only resulted in the development of squamous papillomas with mild dysplasia, following a long latency. Thus, we show the acute sensitivity of the oral mucosa to oncogenic *K*-*ras* in oral tumour development.

## Materials and methods

### Mice and dosing regimes

Mice were housed in accordance with Home Office regulations (UK) and fed a diet of mouse pellets and water *ad libitum*. Mice carrying the *Lox-stop-Lox* (*LSL*)*-K-ras*^*G*12*D*^ allele 8 were kindly provided by David Tuveson (Cancer Research Institute, Cambridge, UK). Mice carrying the tamoxifen-inducible Cre knocked into the *Rosa* locus (*Rosa*^*CreERT*2^) 21 and mice carrying a floxed *LacZ* reporter gene with an internal stop codon knocked into the *Rosa* locus (*Rosa*^*R*26*R*^) 22 were kindly provided by Jos Jonkers (Netherlands Cancer Institute, Amsterdam, The Netherlands). All mice were on a mixed 129/Sv-C57BL/6J background. To achieve somatic expression of oncogenic *K*-*ras*, 8-week-old mice carrying both the *Lox-stop-Lox* (*LSL*)*-K-ras*^*G*12*D*^ and *Rosa*^*CreERT*2^ alleles (LSL-*K*-*ras*^+/*G*12*D*^; *Rosa*^*CreERT*2/*CreERT*2^; hereafter termed *K*-*ras*^+/*G*12*D*^) and wild-type littermate controls (LSL-*K*-*ras*^+/+^; *Rosa*^*CreERT*2/*CreERT*2^; hereafter termed *K*-*ras*^+/+^) were given a single intraperitoneal dose of tamoxifen (4-OHT; Tamoxifen Free Base, MP Biomedicals, Cleveland, OH, USA) dissolved in sterile sunflower oil. Some mice were also left ‘untreated’ to assess spontaneous levels of tumour development (due to ‘leaky’ Cre expression from the *Rosa*^*CreERT*2^ allele). For 5-ethynyl-2′-deoxyuridine (EdU) experiments, mice were dosed intraperitoneally with 0.5 mg of EdU (Invitrogen, Paisley, UK) dissolved in phosphate-buffered saline (pH 7.5) 1 week after tamoxifen treatment and sacrificed 16 h later. For all studies, the mice were examined twice daily for signs of morbidity, at which time they were sacrificed (if not done so earlier) and a full necropsy was performed.

### Genotyping

Genotyping for the *LSL-K-ras*^*G*12*D*^ 8, *Rosa*^*CreERT*2^ 21, and *Rosa*^*R*26*R*^ 22 alleles was performed as described previously. Excision of the stop cassette from the *LSL-K-ras*^*G*12*D*^ allele in tamoxifen-dosed mice was determined using PCR amplification with primers KrasSLRec3A: 5′-GCA GCT AAT GGC TCT CAA AGG-3′ and KrasSLRec3B: 5′-TTG GCT CCA ACA CAG ATG TTC-3′ (62 °C annealing temperature for 5 cycles, followed by 60 °C for 30 cycles). The PCR products were resolved in a 3% agarose gel with the wild-type and activated *K*-*ras*^*G*12*D*^ alleles generating a 196 bp and a 240 bp band, respectively.

### Reverse transcription (RT)-PCR and restriction fragment length polymorphism (RFLP) analysis

Total RNA (1.5 µg) isolated from oral tissue using TRIzol reagent (Invitrogen, Carlsbad, CA, USA) was treated with Turbo Dnase (Ambion, Austin, TX, USA) and reverse-transcribed using the BD Sprint Kit with random hexamers (BD Clontech, Mountain View, CA, USA), according to the manufacturers' instructions. The cDNA was then subjected to PCR and RFLP analysis to distinguish between the wild-type and mutant *K*-*ras* alleles, as previously described 15.

### Histological analysis

All tissues and tumours were fixed in 10% neutral-buffered formalin (NBF) at room temperature over-night. Samples were then transferred to 75% ethanol, embedded in paraffin, sectioned, and stained with haematoxylin and eosin (H&E).

### Immunofluorescence, immunohistochemistry, and β-gal staining

Immunofluorescence analysis was performed on tissue samples that had been embedded and frozen in OCT compound at − 70 °C. Sections were probed with anti-phospho-ERK 1/2, anti-phospho-AKT (#9106 and #4058, Cell Signalling Technology, New England BioLabs, Hertfordshire, UK), and anti-cyclin D1 (RM-9104, Neomarkers, Thermo Scientific, Cheshire, UK). Following incubation with primary antibodies, positive cells were visualized with Alexa Fluor 488-conjugated goat anti-rabbit antibodies for phospho-AKT and cyclin D1, and Alexa Fluor 547-conjugated goat anti-mouse antibodies for phospho-ERK (Invitrogen).

Whole mounts of lip and oral epithelium were prepared as described previously 23. EdU incorporation was detected with a Click chemistry kit (Invitrogen) according to the manufacturer's instructions. Digital images were captured using confocal microscopes (Zeiss LSM 510 META and NIKON Eclipse TE2000-U).

Immunohistochemical analysis was performed on formalin-fixed, paraffin-embedded tissue sections using either rabbit polyclonal anti-β galactosidase antibody (Abcam, Cambridge, UK), rabbit polyclonal anti-cytokeratin 6 (Abcam), rabbit monoclonal anti-cytokeratin 13 (EPR3671; Abcam) or rat anti-mouse Ki-67 (Dako UK Ltd, Ely, UK). Immunohistochemical signal was detected by secondary biotinylated donkey anti-rabbit antibody (Stratech Scientific, Suffolk, UK), followed by a Vector ABC tertiary kit (Vector Laboratories, Burlingame, CA, USA) according to the manufacturer's instructions. All immunohistochemistry was performed on a BondMax machine (Vision Biosystems, Newcastle, UK), according to the manufacturer's instructions, by the Human Research Tissue Bank at Addenbrooke's Hospital (Cambridge, UK).

The raw array CGH data may be found at ftp://ftp. sanger.ac.uk/pub/da1/KRAS_lvdw.

## Results

### Hyperplasia and neoplasia of the oral mucosa in response to expression of *K*-*ras*^*G*12*D*^

**Figure 1:**
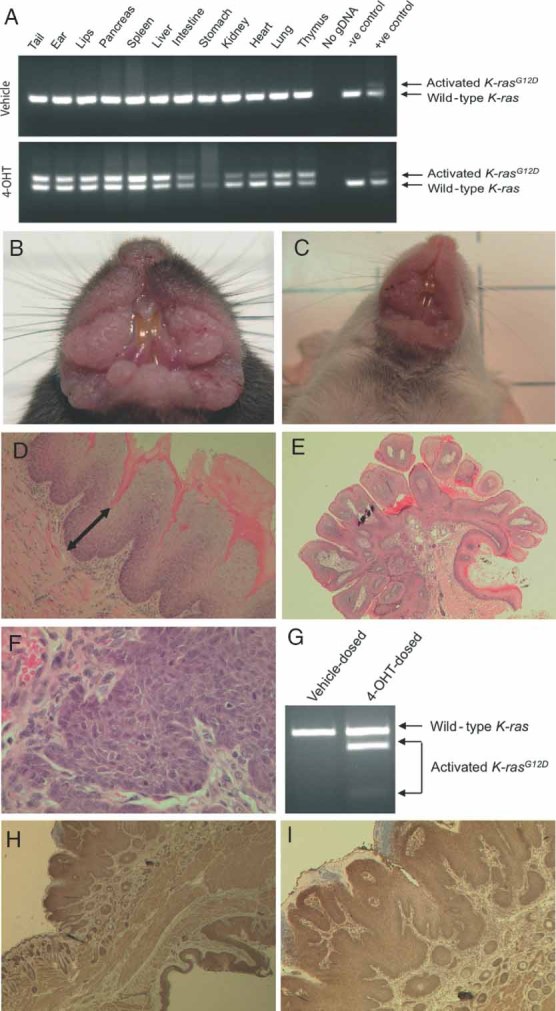
Somatic activation of the *K*-*ras*^*G*12*D*^ allele in mice. (A) PCR analysis of genomic DNA to detect excision of the ‘stop’ cassette in *K*-*ras*^+/*G*12*D*^ mice 1 week after being dosed with vehicle or 1 mg of tamoxifen. The wild-type allele generates a 196 bp product, whereas the activated *K*-*ras*^*G*12*D*^ allele generates a 240 bp product. The negative control was tail gDNA from a *K*-*ras*^+/+^ mouse and the positive control was oral tumour gDNA from a *K*-*ras*^+/*G*12*D*^ mouse. (B, C) Gross appearance of oral tumours that developed in *K*-*ras*^+/*G*12*D*^ mice 14 days after being dosed intraperitoneally with 1 mg of tamoxifen. (D–F) Histological analysis of the oral mucosa following haematoxylin and eosin staining showed squamous hyperplasia (D, see arrow; original magnification × 100), squamous papilloma with dysplasia (E, original magnification × 25), and early invasive squamous cell carcinoma (F, original magnification × 400). All sections shown are representative. (G) RNA expression of wild-type and mutant *K*-*ras* (*K*-*ras*^*G*12*D*^) in lip tissue of mice 14 days after being dosed intraperitoneally with 1 mg of tamoxifen or vehicle. Total RNA was isolated from oral tissue and tumours and reverse-transcribed. The resulting cDNA was PCR-amplified and subjected to *Hind*III digestion as previously described 8. The 448 bp PCR product is digested to generate 300 and 148 bp fragments in the mutant but not the wild-type PCR (as the mutant *K*-*ras*^*G*12*D*^ allele contains a *Hind*III restriction site engineered in exon 1, which is absent in the wild-type allele). (H, I) Immunohistochemical analysis of β-galactosidase expression in the oral cavity of in *K*-*ras*^+/*G*12*D*^; *Rosa*^+/*CreERT*2^; *Rosa*^+/*Rosa*26*R*^ mice dosed with 1 mg of tamoxifen at 8 weeks of age (H, × 25 and I, × 50 original magnification, respectively)

*K*-*ras*^+/*G*12*D*^ mice were given a single intraperitoneal dose of 1 mg of tamoxifen at 8 weeks of age, resulting in activation of the oncogenic *K*-*ras*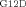, as demonstrated by PCR analysis on DNA extracted from a wide range of tissues 1 week after induction (Figure 1A). Within 14 days of dosing, a marked hyperproliferative phenotype was observed in the oral cavity of *K*-*ras*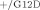 mice, but not *K*-*ras*^+/+^ mice (Figures 1B and 1C). At this time-point, the study was terminated and the mice were sacrificed as they had become progressively malnourished from an inability to feed or drink. Histological analysis of the oral mucosa from these mice showed squamous hyperplasia (Figure 1D), papilloma formation with mild–moderate dysplasia (Figure 1E), and areas of early invasive squamous cell carcinoma (Figure 1F), often with combinations of adjacent squamous hyperplasia, dysplastic papilloma, and focal invasion. The total penetrance of this phenotype (25/25 mice), together with the rapid development of the oral tumours, suggests that few additional mutations or epigenetic changes are required for mutant *K*-*ras*-induced oral squamous hyperplasia and papilloma formation with neoplasia, supported by CGH analysis of tumours from these mice revealing no gross genomic instability (Supporting information, Supplementary Figure 1).

Molecular analysis of oral lesions by RT-PCR and RFLP confirmed that the mutant (activated) *K*-*ras*^*G*12*D*^ allele was being expressed (Figure 1G). In the absence of an antibody specific for the activated form of *K*-ras, we used Rosa26 reporter (*Rosa*^+/*R*26*R*^) mice as a surrogate marker for Cre-mediated activation of *K*-ras, as these mice carry a *loxP*-flanked STOP cassette that prevents expression of a *lacZ* gene; however, when crossed with a mouse line carrying Cre recombinase, the STOP cassette is removed and *lacZ* is expressed in the cells/tissues where Cre is expressed 22. Immunohistochemical analysis of β-galactosidase expression in the oral mucosa of *K*-*ras*^+/*G*12*D*^; *Rosa*^+/*CreERT*2^; *Rosa*^+/*R*26*R*^ mice dosed with 1 mg of tamoxifen at 8 weeks of age showed oral squamous hyperplasia, papilloma formation, and dysplastic papilloma (with irregular basal aspect) with strong β-galactosidase expression in stromal cells as well as in the squamous epithelium (Figures 1H and 1I and Supporting information, Supplementary Figure 2). This shows that there is widespread Cre recombinase activity in the oral mucosa of tamoxifen-treated mice carrying the *Rosa*^*CreERT*2^ allele and thus implies widespread expression of oncogenic *K*-*ras*^*G*12*D*^ in the oral mucosa.

### Widespread hyperplastic changes in multiple tissues in response to expression of *K*-*ras*^*G*12*D*^

At the time of sacrifice (2 weeks post-tamoxifen administration), the *K*-*ras*^*G*12*D*^-expressing mice also showed hyperplastic changes in many other tissues containing moist or frequently abraded squamous mucosa, and occasionally other epithelia, both squamous (anal canal, forestomach, vagina, cervix, paw skin pads) and non-squamous (ovary, endometrium) (Supporting information, Supplementary Figure 3). In addition, hyperplasia was noted in haematopoietic organs (bone marrow, spleen, and liver). However, the lungs showed multiple adenocarcinomas (Supporting information, Supplementary Figure 3). This hyperproliferative and neoplastic phenotype was observed in all the mice examined (*n* = 10), although the relative degree of involvement of each of the tissues varied from mouse to mouse. No other tissues histologically examined (pancreas, kidney, bladder, testes, small intestine, colon, caecum, oesophagus, thymus, heart, salivary gland, brain, eye, ear, and tongue) showed any evidence of hyperplasia or neoplasia. No hyperproliferation of any tissues was observed in tamoxifen-dosed wild-type littermates (*K*-*ras*^+/+^ mice; *n* = 4 mice sacrificed 31 days after dosing).

### Hyperplasia of the oral mucosa occurs in the squamous epithelium of the mucosal surface, hair follicles, and sebaceous glands

**Figure 2:**
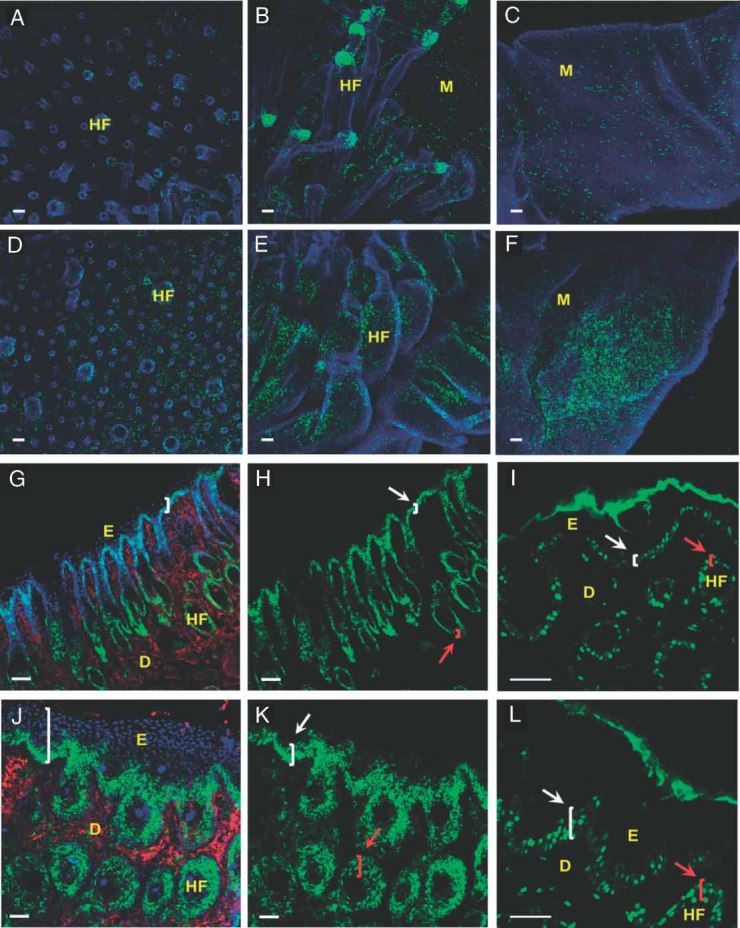
Hyperproliferation of the oral squamous epithelium in *K*-*ras*^*G*12*D*^-expressing mice. (A–F) Whole-mount images. *K*-*ras*^+/*G*12*D*^ mice were dosed intraperitoneally with vehicle (upper panel images) or 1 mg of tamoxifen (lower panel images) and then 1 week later dosed with 0.5 mg of EdU before being sacrificed for tissue collection 16 h later. Confocal image Z stack projections of lip whole-mounts show the outer lip (A, D), mucosa (C, F), and interface space between them (B, E). The whole mounts were stained for EdU (green) and DAPI (blue). (G–L) Immunofluorescence images. *K*-*ras*^+/*G*12*D*^ mice were dosed intraperitoneally with vehicle (upper panels) or 1 mg of tamoxifen (lower panels) and sacrificed 7 days later. Lip cryosections were stained for (G, J) pAKT 473 (green), pERK 1/2 T202/Y204 (red), and DAPI (blue); (H, K) pAKT 473 (green); and (I, L) cyclin D1 (green). D = dermis; E = epidermis; HF = hair follicle. White arrows and brackets indicate the thickness of the epithelium, and red arrows and brackets show the thickness of the hair follicle epithelium. Scale 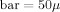
m

To examine more closely the marked proliferative response of the oral mucosa to oncogenic *K*-*ras*, we treated *K*-*ras*^+/*G*12*D*^ mice with 1 mg of tamoxifen (or vehicle) and then 1 week later treated the mice with 0.5 mg of EdU to mark S-phase cells. The mice were sacrificed and tissue samples collected 16 h after EdU dosing. At this time-point, the lips and oral mucosa appeared macroscopically indistinguishable from vehicle-dosed littermates (‘controls’); however, whole-mount preparation of the lips and oral mucosa showed marked hyperplasia compared with the lips and oral mucosa from control animals (Figures 2A–2F). Microscopic examination of the hair-bearing external epithelium of the outer lip (Figures 2A and 2D), wet oral mucosa (Figures 2C and F), and interface between them (Figures 2B and 2E) revealed the hyperproliferative phenotype to be in the surface mucosal epithelium, hair follicles, and sebaceous glands, as shown by the high percentage of EdU-positive cells in these areas.

### Hyperplasia of the squamous epithelium in the oral mucosa signals via the pAKT pathway

**Figure 3:**
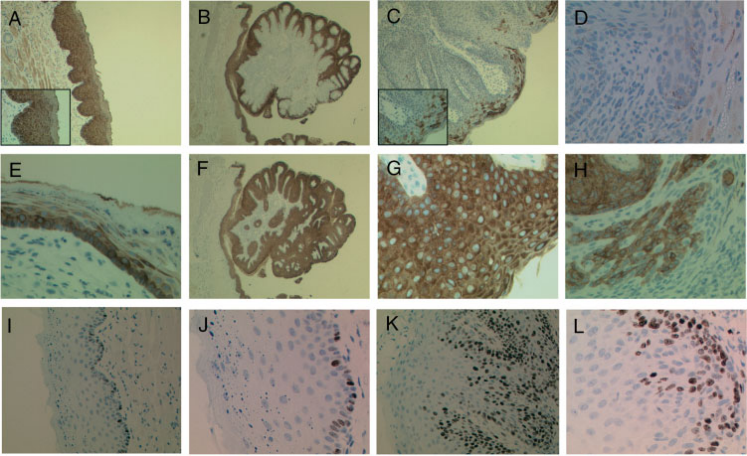
Keratin and proliferation staining in the oral epithelium of dosed *K*-*ras*^*G*12^ mice. Oral epithelium (lips) were immunostained for cytokeratin 13 (A–D), cytokeratin 6 (E–H), or Ki-67 (I–L) in *K*-*ras*^+/*G*12*D*^ mice dosed either with vehicle (A, E, I, J) or with 1 mg of tamoxifen (B–D, F–H, K, L) and collected 14 days later. Original magnification: × 25 (B, C, F); × 100 (A); × 200 (I, K); × 400 (A inset, C inset, D, E, G, H, J, L)

Several Ras effector pathways, including the Raf/MEK/ ERK (MAPK) and PI3K/AKT kinase cascades, promote cell proliferation, differentiation, and survival 24. To establish the mechanism(s) by which activated *K*-*ras* could be causing hyperproliferation in the oral mucosa, we stained cryosections from 1 mg tamoxifen-dosed *K*-*ras*^+/*G*12*D*^ mice and vehicle-dosed controls (1 week after dosing) with antibodies to known downstream targets of Ras signalling, specifically phosphorylated AKT (pAKT) and Erk (pERK) (Figures 2G–2L). pAKT was detected in the hair follicles and basal layers of surface squamous epithelium in both induced and vehicle-treated mice (Figures 2G and 2J). However, the undosed mice showed positive staining in 1–3 layers of cells (Figure 2H), whereas in the dosed mice pAKT was typically detectable in 3–6 layers of cells in the epithelium (Figure 2K), with little positivity in the stromal cells. In contrast, pERK staining was mainly observed in the stromal cells of the dermis and not in the squamous epithelium of the surface or the hair follicles (Figures 2G and 2J). In corroboration that this oral cavity hyperproliferation is taking place downstream of *K*-ras signalling, the hair follicles and basal layers of the squamous epithelium also strongly immunostained for cyclin D1 expression (Figures 2I and 2L) and the proliferation markers EdU and Ki-67 (Figures 2, 3K, and 3L).

Although the most impressive immunofluorescent evidence for increased pAKT signalling occurred in the hair-bearing skin of the outer lip, overall there is evidence of altered proliferation (increased EdU and Ki-67 staining) with cyclin D1 expression in the squamous epithelium of the wet oral mucosa, which is the cell type giving rise to the hyperplasia–papilloma–dysplasia–carcinoma sequence in this model. Thus, pAKT seems to be more active in mutant *K*-*ras*-expressing squamous epithelium, probably contributing to the hyperproliferative phenotype. Indeed, increased expression of pAKT has been reported in ameloblastic tumours (a benign odontogenic neoplasm which can undergo malignant transformation) 25 and benign neoplasms of the salivary gland (pleomorphic adenomas and myoepitheliomas) 26.

### Hyperplastic and neoplastic epithelium shows evidence of aberrant differentiation

The oral squamous epithelium is a self-renewing tissue with terminal differentiation, involving changes in the expression of cytokeratins, which can be studied as major differentiation markers of stratified epithelia. Cytokeratins have also been associated with different stages of tumour progression in human oral tumours 27, 28. To determine whether the oral tumours that developed 14 days after 1 mg tamoxifen treatment of *K*-*ras*^+/*G*12*D*^ mice exhibited changes in the differentiation patterns, we examined the expression patterns of key cytokeratins expressed in oral epithelia. Specifically, we studied cytokeratin 13 (CK13), found consistently in the suprabasal layers of normal squamous epithelia of the oral cavity with decreased/absent expression in squamous cell dysplasia/carcinoma 29–31, and cytokeratin 6 (CK6), not normally expressed above the basal layer of normal squamous epithelium, but rapidly induced in suprabasal (post-mitotic) cells during conditions of hyperproliferation 32. We found that CK13 expression was decreased from full thickness expression in normal oral squamous epithelium (Figure 3A) to only a few superficial cell layers of expression in oral papillomas (Figure 3B), and to only the most differentiated parts (the superficial or upper epithelium) of hyperplastic and dysplastic squamous epithelium (Figure 3C).

There was no detectable CK13 expression in the invasive tongues and clusters of squamous cell carcinoma (Figure 3D). In contrast, CK6 expression was broadened from the basal and first suprabasal layers seen in normal oral squamous epithelium (Figure 3E) to almost all cell layers in oral papillomas (Figure 3F), and there was expression throughout the full thickness of the hyperplastic and dysplastic squamous epithelium (Figure 3G). High expression was also found in invasive tongues and clusters in the squamous cell carcinoma (Figure 3H). These changes were found in all tamoxifen-dosed *K*-*ras*^+/*G*12*D*^ mice (*n* = 7), but not in tamoxifen-dosed *K*-*ras*^+/+^ (*n* = 5) or vehicle-dosed *K*-*ras*^+/*G*12*D*^ (*n* = 5) control mice. Similar changes in cytokeratin expression patterns were reported in the oral tumours (benign squamous papillomas) from *LSL-K-ras*^*G*12*D*^; K5.Cre*PR1 mice dosed with RU486 in the oral cavity 15, and have been observed in human oral neoplasms 27, suggesting that changes in differentiation markers in the oral tumours that developed in *K*-*ras*^+/*G*12*D*^ mice resemble those observed in human patients.

In addition, in keeping with the EdU results from mice collected 7 days after exposure to tamoxifen (Figures 2A–2F), oral mucosa from tamoxifen-dosed *K*-*Ras*^+/*G*12*D*^ mice collected 14 days after dosing showed an increased number of squamous epithelial cells in several suprabasal layers staining positively for the cell proliferation marker Ki-67 (Figures 3K and 3L), but no stromal cell positivity, compared with vehicle-dosed controls that showed only basal layer epithelial positivity (Figures 3I and 3J).

### Effects of using low doses of tamoxifen

**Figure 4:**
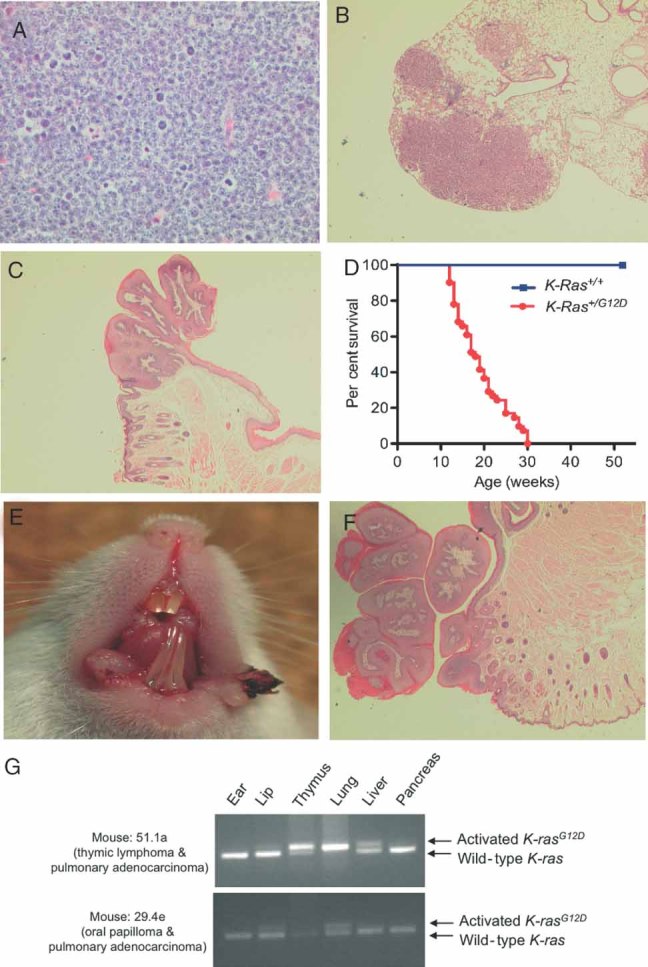
The effect of low Cre expression. Histological analysis of haematoxylin and eosin-stained tissues from *K*-*ras*^+/*G*12*D*^ mice dosed intraperitoneally with 1–10 µg of tamoxifen, including (A) a thymic lymphoma (original magnification × 400), (B) multiple adenocarcinomas of the lung (original magnification × 25), and (C) a lip squamous papilloma with dysplasia (original magnification × 25). (D) Survival of undosed *K*-*ras*^+/+^ and *K*-*ras*^+/*G*12*D*^ mice. The median survival for *K*-*ras*^+/*G*12*D*^ mice was 18–20 weeks. Log-rank (Mantel–Cox) test: *p* > 0.0001. (E) Representative example of the gross appearance of oral tumours that ‘spontaneously’ developed in undosed *K*-*ras*^+/*G*12*D*^ mice. (F) Histological analysis of haematoxylin and eosin-stained section representative of the ‘spontaneous’ oral papillomas observed (original magnification × 25). (G) PCR analysis of Cre-mediated removal of the floxed ‘stop’ cassette (and hence expression of the activated *K*-*ras*^*G*12*D*^) on gDNA from selected tissues of *K*-*ras*^+/*G*12*D*^ mice that spontaneously developed tumours. Mouse 51.1a, which developed a thymic lymphoma and multiple pulmonary adenocarcinomas, showed activated *K*-*ras*^*G*12*D*^ in the thymus, lung, and liver. Mouse 29.4e, which developed an oral papilloma and multiple pulmonary adenocarcinomas, showed activated *K*-*ras*^*G*12*D*^ in the lip and lung

When lower doses of tamoxifen were administered (1–10 µg), to activate oncogenic *K*-*ras* in fewer cells, thymic lymphomas (Figure 4A) and pulmonary adenocarcinomas (Figure 4B) were observed in 100% of the *K*-*ras*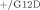 mice within 14–40 weeks post-treatment (7/7 of 10 µg-dosed mice and 5/5 of 1 µg-dosed mice) but never in the tamoxifen-treated *K*-*ras*^+/+^ controls (0/10). One mouse treated with low-dose tamoxifen, however, developed a macroscopic ‘outgrowth’ on the lip/oral mucosa, which histopathologically presented as a squamous papilloma with only a mild degree of dysplasia and no evidence of invasion, with no adjacent hyperplasia of the surrounding squamous epithelium (Figure 4C).

Similarly, sporadic activation of *K*-*ras*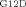 (due to the *Rosa*^*CreERT*2^ allele producing low levels of background recombination in the absence of the inducer 21) resulted in decreased survival of untreated *K*-*ras*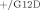 mice (Figure 4D), with mice presenting with pulmonary adenocarcinomas (33/33 cases, 100%) and leukaemia/lymphoma (27/33 cases, 81%). In addition, some mice (8/33, 24%) also developed macroscopic ‘outgrowths’ on their lips and oral mucosa (Figure 4E). These were similar to the lesion shown in Figure 4C, appearing as a single papillomatous outgrowth with only a mild degree of dysplasia and no adjacent hyperplasia of the surrounding squamous epithelium (Figure 4F), and were markedly different to the large areas of hyperplasia and papillomas with dysplasia (with some areas of focal invasion) seen in the *K*-*ras*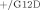 mice dosed with 1 mg of tamoxifen (Figures 1B–1E). In addition, some mice also showed variable degrees of proliferation in the anal squamous epithelium (9/33, 27%), with the most severe cases presenting with anal prolapse. In contrast, mice carrying wild-type alleles at the *K*-*ras* locus (*K*-*ras*^+/+^ mice) never developed tissue hyperplasia or tumours (12/12 mice) within 12 months. These results suggest that acute expression of *K*-*ras*^*G*12*D*^ throughout the abraded and wet mucosal squamous epithelium has a profound field effect on these tissues that differs from the phenotype observed when isolated cells or clones of cells stochastically express *K*-*ras*^*G*12*D*^. Interestingly, array comparative genome hybridization (aCGH) analysis of oral tissue from the spontaneously developed tumours (papillomas; *n* = 3) did not show any more genomic instability than that of the induced tumours (*n* = 5) (Supporting information, Supplementary Figure 1).

PCR amplification of genomic DNA from spontaneously developed tumours in these *K*-*ras*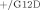 mice showed the presence of the recombined *K*-*ras*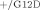 allele (Figure 4G). Interestingly, however, some tissues, such as the liver, showed the presence of the recombined *K*-*ras*^*G*12*D*^ allele but no histopathological abnormality (Figure 4G). Conversely, some tissues, such as the pancreas, did not show the presence of the activated *K*-*ras*^*G*12*D*^ allele (Figure 4G), which explains why tumours were never observed in this tissue 33.

## Discussion

Oral cancer has a high mortality and morbidity, with a 5-year survival rate for patients of ∼50% 34. This high lethality is ascribed to the advanced state of malignancy at the time of detection, as when detected early they can be cured in 80–90% of cases. Thus, understanding the molecular mechanisms involved in initiation and progression to malignancy of oral cancer may help earlier detection to improve the prognosis of the disease and the development of novel therapeutic strategies. Around 5% of human oral cancers have mutated *K*-*ras*, but its role as a driver mutation is uncertain. To this end, we developed a mouse model of widespread somatic expression of oncogenic *K*-*ras* in the adult mouse by crossing *LSL-K-ras*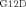 mice with tamoxifen-inducible *Rosa*^*CreERT*2^ mice. Dosing of the offspring with 1 mg of tamoxifen resulted in the development of a marked hyperproliferative and neoplastic phenotype in the oral cavity in *K*-*ras*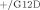 mice (but not *K*-*ras*^+/+^ mice) within 14 days of treatment. This phenotype involved the lips and oral mucosa, showing a sequence of squamous hyperplasia followed by squamous papilloma with dysplasia, in which some areas progressed to early invasive squamous cell carcinoma (Figure 1). The total penetrance of this phenotype, together with the rapid development of the oral tumours, suggests that few additional mutations or epigenetic changes are required for *K*-*ras*-induced oral squamous hyperplasia and dysplastic papilloma formation in mice. Previous studies have shown that mutant *K*-*ras* expression in mice (using *K*-*ras4b*^*G*12*D*^, *K*-*ras*^*G*12*D*^ or *K*-*ras*^*V*12^ alleles) is sufficient to initiate lung adenomas and adenocarcinomas 7, 9, 10, and pancreatic tumours 33 with few additional genetic or epigenetic alterations.

Although our primary interest was in characterizing the oral hyperproliferative and neoplastic phenotype observed in these *K*-*ras*^*G*12*D*^-expressing mice, other hyperplastic and neoplastic pathological abnormalities were also observed. Hyperproliferation was observed in sites of wet or frequently abraded squamous mucosa in addition to the mouth, including the anus, forestomach, vagina/cervix, and paw skin pads. Other studies have also recorded the presence of squamous papillomas in similar tissues (ear and snout skin, anal and vulvo-vaginal squamous mucosa, oesophageal and forestomach squamous epithelium 9, 11, 18), as well as lymphoma/leukaemia 9, 18, and lung adenoma/adenocarcinomas 8–11, 18 in oncogenic *K*-*ras*-expressing mice. In addition, *K*-*RAS* is one of the most commonly mutated oncogenes in human lung cancer 35, and oncogenic *RAS* mutations have also been detected in haematological malignancies 36, 37.

Interestingly, however, some tissues consistently showed the presence of the activated *K*-*ras*^*G*12*D*^ allele, yet never developed any pathological abnormality, such as the liver, suggesting that expression of oncogenic *K*-*ras* at physiological levels is not sufficient to induce unscheduled proliferation in certain cell types, and mutated *K*-*ras* does not seem to initiate tumourigenesis in certain tissue types, as shown in the liver here and previously 12. Conversely, some tissues did not show the presence of the activated *K*-*ras*^*G*12*D*^ allele, such as the pancreas, which explains why tumours of this tissue were never observed despite being seen in other studies 33 and oncogenic *K*-*RAS* being a known inducer of pancreatic neoplasia (over 90% of cases of pancreatic cancer show mutations in *K*-*RAS* 38).

The reason why these animals develop hyperplasia and neoplasia in the lips and oral mucosa more readily than in other tissues (apart from the lung) may be partly due to the fact that the oral squamous mucosa has high rates of proliferation due to the necessity to maintain a tissue which is frequently exposed to mild abrasion or chewing-related trauma. Furthermore, the pre-existing signalling network status of the oral squamous epithelium may be particularly sensitive to activation of the *K*-Ras/PI3K/AKT pathway, making these cells more prone to tumour development compared with other tissues. The stromal cells under the oral epithelium also showed Cre-mediated activation using the β-*gal* reporter system, indicating mutant *K*-*ras* activation in these cells as well, corroborated by pERK activation detected in these cells by immunofluorescence. Therefore, cross-talk between *K*-*ras*^*G*12*D*^-expressing squamous epithelium and *K*-*ras*^*G*12*D*^-expressing adjacent stroma may have cooperated to generate rapidly developing hyperplasia and neoplasia manifested in the squamous epithlium. Oncogenic *K*-*ras*^*G*12*D*^-induced proliferation in the oral cavity has previously been reported in two studies. In the first study, ∼30% of *LSL-K-ras*^+/*G*12*D*^; *Mx1*^+/*Cre*^ mice developed squamous papillomas in the oral mucosa 18. However, there was no further analysis of these tumours as this was not relevant to the study. In the second study, *LSL-K-ras*^*G*12*D*^; K5.Cre*PR1 mice dosed with RU486 in the oral cavity (to induce *K*-*ras*^*G*12*D*^ expression under the control of the K5 promoter) developed exophytic papillomas of the oral mucosa, tongue, and palate 16–24 weeks after exposure 15. Histologically, these tumours were classified as benign, showing marked hyperplasia and up to severe dysplasia, but with no evidence of invasion into the submucosal tissue.

The phenotype observed by Caulin *et al* 15 is similar to that observed in our sporadic study in which some of the mice (24%) developed squamous papillomas with only a mild degree of dysplasia, but no invasion. The isolated nature of these tumours and the absence of hyperplasia in the surrounding normal squamous epithelium suggest that these tumours have originated as a result of clonal expansion of a single cell expressing an activated *K*-*ras*^*G*12*D*^ allele. In contrast, mice with widespread *K*-*ras*^*G*12*D*^ expression in the oral mucosal epithelial and stromal cells showed acute and generalized hyperproliferation of the oral squamous epithelium that rapidly progressed to dysplastic papillomas with focal invasion. This raises the possibility that mutant *K*-*ras* expression in a field of epithelial and stromal cells does not just alter the behaviour of individual cells, but may have a ‘community effect’.

Thus, the data presented here show that tumour development induced by oncogenic *K*-*ras*^*G*12*D*^ is highly dependent on the cell type, with oral squamous epithelium displaying acute sensitivity to oncogenic *K*-*ras*. For the oral cavity, we have shown that oncogenic *K*-*ras* is an important inducer of tumourigenesis that functions via the pAKT pathway to initiate squamous hyperplasia, with progression to papilloma formation with dysplasia and ultimately invasive squamous carcinoma, producing lesions with differentiation characteristics resembling those shown in human oral neoplasms. Furthermore, if low doses of tamoxifen are used, or spontaneous recombination occurs, the development of squamous papillomas with dysplasia after a long latency may allow the testing of anti-RAS preventative and therapeutic agents.

## SUPPORTING INFORMATION ON THE INTERNET

The following supporting information may be found in the online version of this article.

**Figure S1.** Array CGH analysis of chromosomal alterations in the oral tumours from *K*-*ras*^*G*12*D*^ mice.

**Figure S2.** Immunohistochemical analysis of β-galactosidase expression in the oral cavity of *K*-*ras* mice.

**Figure S3.** Widespread tissue hyperproliferation in *K*-*ras*^*G*12*D*^-expressing mice.

## References

[1] Bos JL (1989). *ras* oncogenes in human cancer. A review. Cancer Res.

[2] Young A, Lyons J, Miller AL (2009). Ras signaling and therapies. Adv Cancer Res.

[3] Adjei AA (2001). Blocking oncogenic Ras signaling for cancer therapy. J Natl Cancer Inst.

[4] Das N, Majumder J, DasGupta UB (2000). Ras gene mutations in oral cancer in eastern India. Oral Oncol.

[5] Brookman KC, Schönleben F, Qiu W (2010). Mutational analyses of the *BRAF, KRAS*, and *PIK3CA* genes in oral squamous cell carcinoma. Oral Surg Oral Med Oral Pathol Oral Radiol Endodontol.

[6] Shin KH, Bae SD, Hong HS (2010). miR-181a shows tumor suppressive effect against oral squamous cell carcinoma cells by downregulating *K*-ras. Biochem Biophys Res Commun.

[7] Fisher GH, Wellen SL, Klimstra D (2001). Induction and apoptotic regression of lung adenocarcinomas by regulation of a *K*-Ras transgene in the presence and absence of tumour suppressor genes. Genes Dev.

[8] Jackson EL, Willis N, Mercer K (2001). Analysis of lung tumour initiation and progression using conditional expression of oncogenic *K*-ras. Genes Dev.

[9] Johnson L, Mercer K, Greenbaum D (2001). Somatic activation of the *K-ras* oncogene causes early onset lung cancer in mice. Nature.

[10] Meuwissen R, Linn SC, van der Valk M (2001). Mouse model for lung tumourigenesis through Cre/lox controlled sporadic activation of the *K*-Ras oncogene. Oncogene.

[11] Guerra C, Mijimolle N, Dhawahir A (2003). Tumour induction by an endogenous *K*-ras oncogene is highly dependent on cellular context. Cancer Cell.

[12] Luo F, Brooks DG, Ye H (2007). Conditional expression of mutated *K**ras* accelerates intestinal tumourigenesis in Msh2-deficient mice. Oncogene.

[13] Luo F, Hamoudi R, Brooks DG (2007). Stem cell gene expression changes induced specifically by mutated *K**ras*. Gene Expr.

[14] Luo F, Brooks DG, Ye H (2009). Mutated *K**ras*^*Asp*12^ promotes tumourigenesis in *Apc*^*Min*/+^ mice more in the large than small intestines, with synergistic effects between *K**ras* and *Wnt* pathway. Int J Exp Pathol.

[15] Caulin C, Nguyen T, Longley MA (2004). Inducible activation of oncogenic *K*-ras results in tumour formation in the oral cavity. Cancer Res.

[16] Tuveson DA, Shaw AT, Willis NA (2004). Endogenous oncogenic *K*-ras(G12D) stimulates proliferation and widespread neoplastic and developmental defects. Cancer Cell.

[17] Braun BS, Tuveson DA, Kong N (2004). Somatic activation of oncogenic Kras in hematopoietic cells initiates a rapidly fatal myeloproliferative disorder. Proc Natl Acad Sci U S A.

[18] Chan IT, Kutok JL, Williams IR (2004). Conditional expression of oncogenic *K*-ras from its endogenous promoter induces a myeloproliferative disease. J Clin Invest.

[19] Izeradjene K, Combs C, Best M (2007). Kras(G12D) and Smad4/Dpc4 haploinsufficiency cooperate to induce mucinous cystic neoplasms and invasive adenocarcinoma of the pancreas. Cancer Cell.

[20] Vincent DF, Yan KP, Treilleux I (2009). Inactivation of TIF1gamma cooperates with Kras to induce cystic tumours of the pancreas. PLoS Genet.

[21] Hameyer D, Loonstra A, Eshkind L (2007). Toxicity of ligand-dependent Cre recombinases and generation of a conditional Cre deleter mouse allowing mosaic recombination in peripheral tissues. Physiol Genomics.

[22] Soriano P (1999). Generalized lacZ expression with the ROSA26 Cre reporter strain. Nature Genet.

[23] Braun KM, Niemann C, Jensen UB (2003). Manipulation of stem cell proliferation and lineage commitment: visualisation of label-retaining cells in wholemounts of mouse epidermis. Development.

[24] Vojtek AB, Der CJ (1998). Increasing complexity of the Ras signaling pathway. J Biol Chem.

[25] Kumamoto H, Ooya K (2007). Immunohistochemical detection of phosphorylated Akt, PI3K, and PTEN in ameloblastic tumours. Oral Dis.

[26] Marques YM, de Lima Mde D, de Melo Alves Sde M (2008). Mdm2, p53, p21 and pAKT protein pathways in benign neoplasms of the salivary gland. Oral Oncology.

[27] van der Velden LA, Manni JJ, Ramaekers FC (1999). Expression of intermediate filament proteins in benign lesions of the oral mucosa. Eur Arch Otorhinolaryngol.

[28] Bloor BK, Seddon SV, Morgan PR (2001). Gene expression of differentiation-specific keratins in oral epithelial dysplasia and squamous cell carcinoma. Oral Oncol.

[29] Ohkura S, Kondoh N, Hada A (2005). Differential expression of the keratin-4, -13, -14, -17 and transglutaminase 3 genes during the development of oral squamous cell carcinoma from leukoplakia. Oral Oncol.

[30] Yanagawa T, Yoshida H, Yamagata K (2007). Loss of cytokeratin 13 expression in squamous cell carcinoma of the tongue is a possible sign for local recurrence. J Exp Clin Cancer Res.

[31] Schaaij-Visser TB, Graveland AP, Gauci S (2009). Differential proteomics identifies protein biomarkers that predict local relapse of head and neck squamous cell carcinomas. Clin Cancer Res.

[32] Freedberg IM, Tomic-Canic M, Komine M (2001). Keratins and the keratinocyte activation cycle. J Invest Dermatol.

[33] Hingorani SR, Petricoin EF, Maitra A (2003). Preinvasive and invasive ductal pancreatic cancer and its early detection in the mouse. Cancer Cell.

[34] Pugliano FA, Piccirillo JF, Zequeira MR (1999). Clinical-severity staging system for oral cavity cancer: five-year survival rates. Otolaryngol Head Neck Surg.

[35] Riely GJ, Marks J, Pao W (2009). KRAS mutations in non-small cell lung cancer. Proc Am Thorac Soc.

[36] Beaupre DM, Kurzrock R (1999). RAS and leukemia: from basic mechanisms to gene-directed therapy. J Clin Oncol.

[37] Reuter CW, Morgan MA, Bergmann L (2000). Targeting the Ras signaling pathway: a rational, mechanism-based treatment for hematologic malignancies?. Blood.

[38] Downward J (2003). Targeting RAS signalling pathways in cancer therapy. Nature Rev Cancer.

[39] Gentleman RC, Carey VJ, Bates DM (2004). Bioconductor: open software development for computational biology and bioinformatics. Genome Biol.

[40] van de Wiel MA, Kim KI, Vosse SJ (2007). CGHcall: an algorithm for calling aberrations for multiple array CGH tumor profiles. Bioinformatics.

[41] Venkatraman ES, Olshen AB (2007). A faster circular binary segmentation algorithm for the analysis of array CGH data. Bioinformatics.

