# Supplementary figures and images for: Acute sensitivity of the oral mucosa to oncogenic K-ras
Source: J Pathol. 2011 Mar 7;224(1):22–32. doi: 10.1002/path.2853 (PMC3627303; doi:10.1002/path.2853)

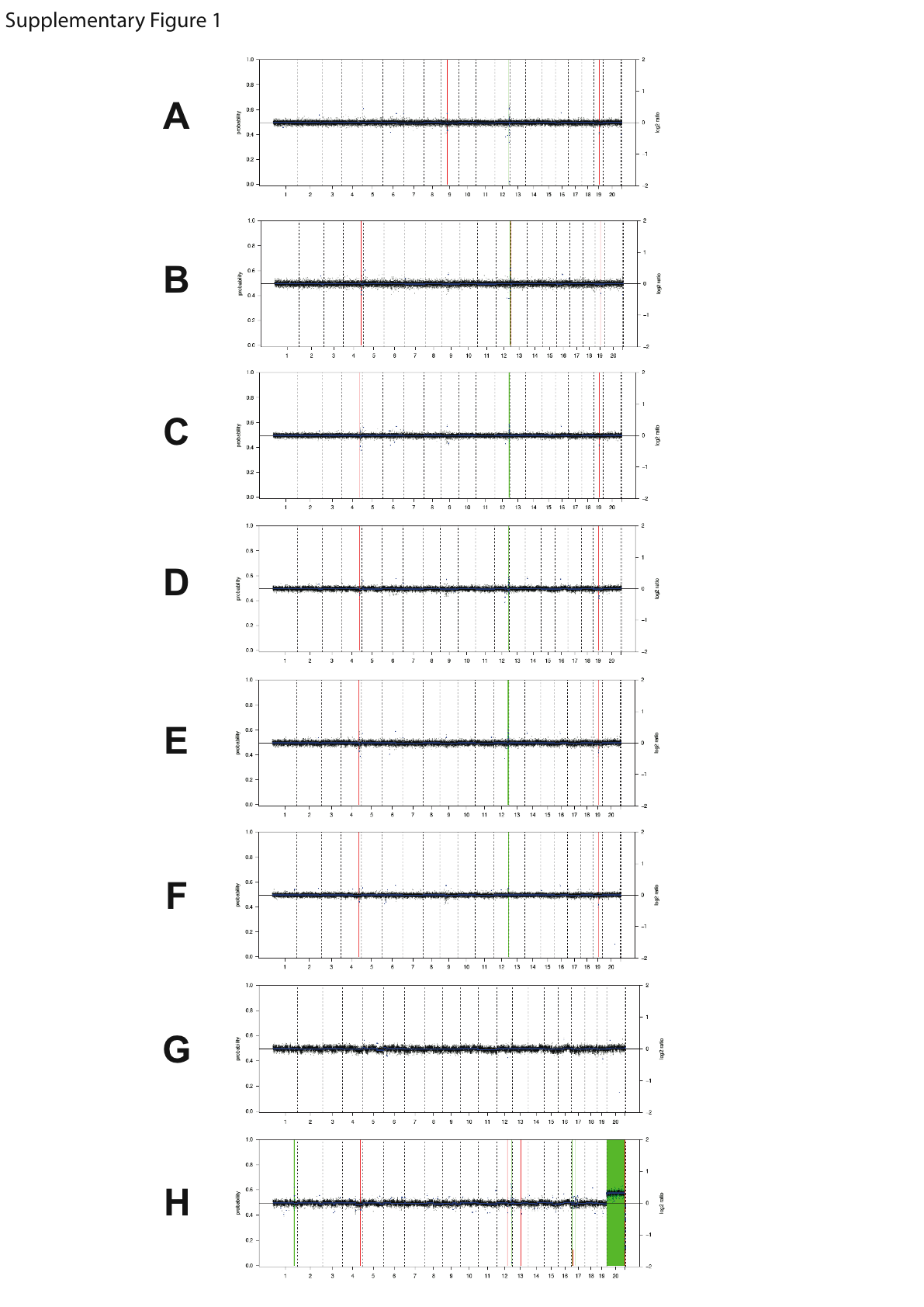

Supplement: Supplementary file 1 [file path0224-0022-SD1.tif]

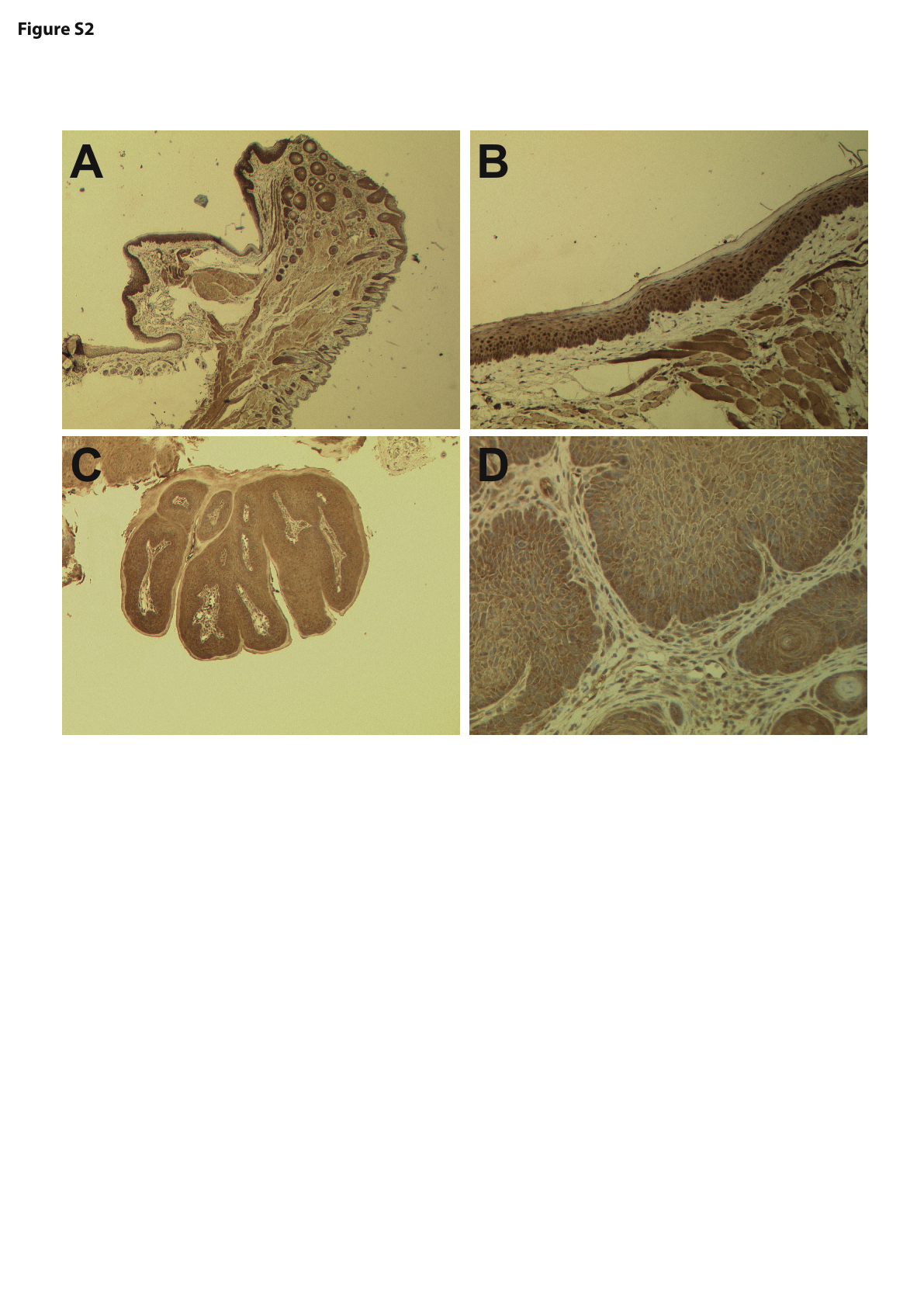

Supplement: Supplementary file 2 [file path0224-0022-SD2.jpg]

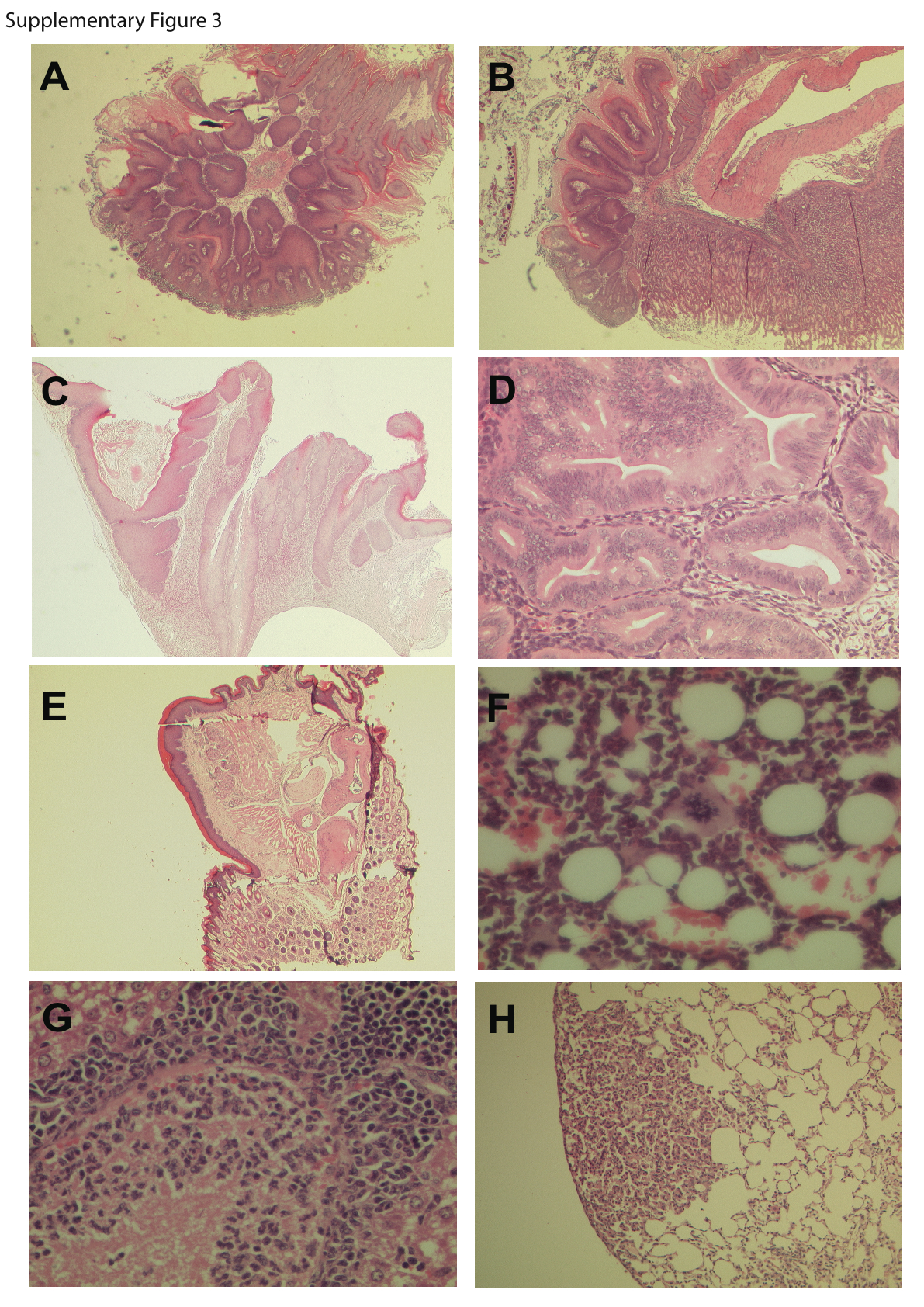

Supplement: Supplementary file 3 [file path0224-0022-SD3.jpg]
