# Supplementary material for: Acute sensitivity of the oral mucosa to oncogenic K-ras
Source: J Pathol. 2011 Mar 7;224(1):22–32. doi: 10.1002/path.2853 (PMC3627303; doi:10.1002/path.2853)
Supplement: Supplementary file 4 [file path0224-0022-SD4.doc]

**Supporting information**

**Supplementary figure legends**

**Figure S1. Array CGH analysis of chromosomal alterations in the oral tumours from *K*-*ras^G12D^* mice.** Array CGH analysis of DNA copy number changes in the spontaneous (n=3, **A-C**) and tamoxifen-induced (n=5, **D-H**) oral tumours from *K-ras^+/G12D^* mice was performed using the Mouse Genome CGH Microarray 244A (Agilent Technologies, Cheshire, UK). Log_2_ratio data were initially pre-processed to remove probes occurring multiple times on the array, resulting in a set of 233,212 unique data points for each sample. The Bioconductor [39] packages CGHcall (version 2.8.0) [40] and DNAcopy (version 1.20.0) [41] were combined to call genomic regions that had undergone gains and losses. The data was pre-processed and normalized using CGHcall with default settings.  Segmentation of the data into smoothed observations of the log-ratios was performed using DNAcopy, and default settings used except that localised change-points were removed using the parameter "undo.splits='sdundo'". Gains and losses were called on the segmented data using CGHcall. No statistically significant changes were observed between the two cohorts.

**Figure S2. Immunohistochemical analysis of β-galactosidase expression in the oral cavity of K-*ras* mice**. K-ras^+/G12D^ mice were intercrossed with *Rosa26R* mice (which carry a *LacZ* reporter gene with an internal ATOP codon flanked by *Lox*P sites [22]) and the offspring dosed with 1 mg tamoxifen at 8 weeks of age, and their lips collected 3 weeks later. (**A, B**) *K-ras^+/+^; Rosa26R* mice show normal lip histology (thin outer skin and inner lip surface squamous mucosa, but no abnormalities; x25 and x100 magnification, respectively). (**C,** **D**) *K-ras*^+/G12D^; *Rosa26R* mice developed swollen lips and the histology shows squamous papilloma formation and dysplastic papilloma with irregular basal aspect (x50 and x400 magnification, respectively). Note: β-galactosidase expression (and thus recombination) is seen in stromal cells as well as in the squamous epithelium.

**Figure S3. Widespread tissue hyperproliferation in K-*ras^G12D^*-expressing mice.** Histological analysis of haematoxylin-eosin stained tissues from *K-ras^+/G12D^* mice 14 days after being i.p. dosed with 1 mg tamoxifen showed evidence of hyperproliferation, including **(A)** anal squamous papilloma with dysplasia (x25 magnification), **(B)** diffuse squamous papilloma of forestomach and glandular hyperplasia (x25 magnification), **(C)** cervix and vagina showing squamous hyperpasia with some dysplasia and early papilloma formation (x25 magnification), **(D)** complex atypical hyerplasia of endometrium (x200 magnification), **(E)** squamous hyperplasia of the paw (x25 magnification), **(F)** hypercellular bone marrow (x400 magnification), **(G)** leukaemia in the liver (x400 magnification), and **(H)** lung adenocarcinoma (x200 magnification). All sections shown are representative.

**References**

**(Note: reference numbers correspond to reference list in main article)**

22. [Soriano P](http://www.ncbi.nlm.nih.gov/pubmed?term="Soriano P"%5BAuthor%5D). Generalized lacZ expression with the ROSA26 Cre reporter strain. [*Nature Genet*](javascript:AL_get(this, 'jour', 'Nat Genet.');) 1999; **21:** 70–71.

39. Gentleman RC, Carey VJ, Bates DM, *et al*. [Bioconductor: open software development for computational biology and bioinformatics.](http://www.ncbi.nlm.nih.gov/pubmed/15461798) *Genome Biol* 2004; **5:** R80.

40. van de Wiel MA, Kim KI, Vosse SJ, *et al*. [CGHcall: an algorithm for calling aberrations for multiple array CGH tumor profiles](http://bioinformatics.oxfordjournals.org/cgi/reprint/btm030?ijkey=nWC7sqXpBTppcnv&keytype=ref). *Bioinformatics* 2007; **23**: 892–894.

41. Venkatraman ES, Olshen AB. A faster circular binary segmentation algorithm for the analysis of array CGH data. *Bioinformatics* 2007; **23**: 657–663.
